# Four 3D coordination polymers based on layers with single *syn*–*anti* carboxylate bridges: synthesis, structures, and magnetic properties†

**DOI:** 10.1039/c8ra01900b

**Published:** 2018-04-16

**Authors:** Wei Gao, Feng Liu, Xiu-Mei Zhang, Jie-Ping Liu, Qing-Yu Gao

**Affiliations:** College of Chemical Engineering China University of Mining and Technology Xuzhou Jiangsu 221116 China gaoqy@cumt.edu.cn; College of Chemistry and Materials Science, Huaibei Normal University Anhui 235000 China zhangxiumeilb@126.com

## Abstract

Four novel coordination polymers (CPs) based on a new 4-(3,5-dicarboxylphenyl) picolinic acid ligands (H_3_L), [M_3_(L)_2_(H_2_O)_6_]·4H_2_O (M_3_ = Mn_3_, 1; Co_3_, 2; Ni_3_, 3, Co_1.01_Ni_1.99_, 4), have been hydrothermally synthesized, and structurally and magnetically characterized. In these isomorphous CPs, octahedrally coordinated metal ions are linked by the single *syn*–*anti* carboxylate bridge (μ-COO) to give linear trinuclear motifs. The motifs are connected through the other single *syn*–*anti* carboxylate bridge (μ-COO) to give a 2D (4,4) layer, and the layers are interlinked by the L ligands into 3D frameworks. Magnetic measurement indicates that antiferromagnetic interactions between metal ions are mediated through the single *syn*–*anti* carboxylate bridges in 1 and 2, while the same carboxylate bridges in 3 transmit ferromagnetic couplings. The bimetallic CP 4 shows interesting complicated magnetic behaviors due to the competition effect of Co(ii) and Ni(ii) ions.

## Abstract 2

Four 3D carboxylate-bridged metal(II) compounds were prepared. Mn(II) and Co(II) compounds show AFM interactions, while FM coupling is found in Ni(II) compound. The bimetallic shows interesting competition effect of FM and AFM interactions.

## Graphical abstract

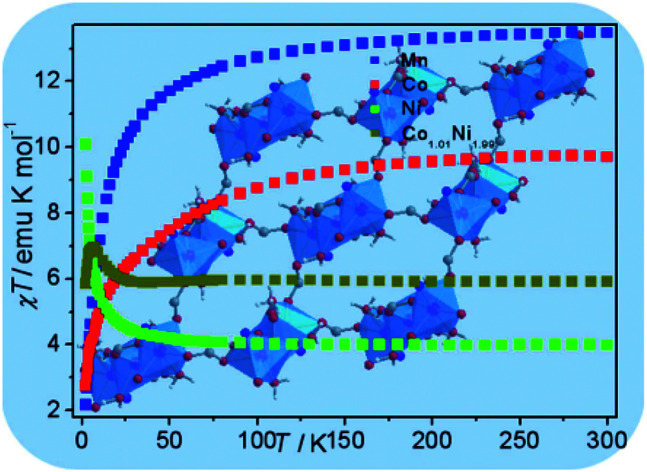

## Introduction

**Scheme 1:**
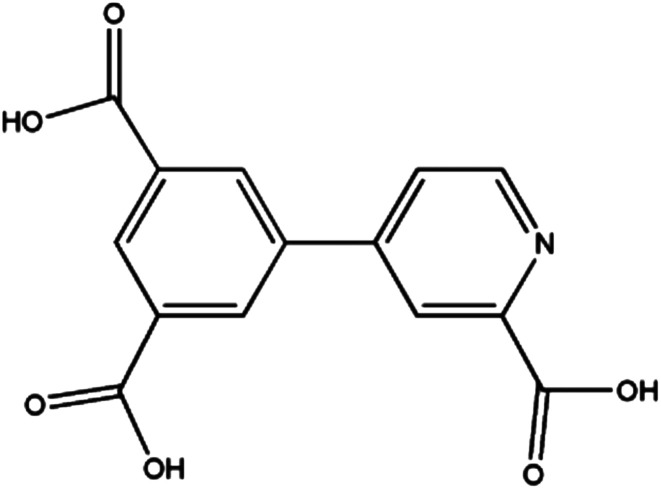
Structures of 4-(3,5-dicarboxylphenyl) picolinic acid (H_3_L) ligand.

Research efforts on metal–organic frameworks (MOFs) or coordination polymers (CPs) have disclosed great versatile multifunctional materials with fascinating structures and promising properties.^1–3^ Such multifunctional materials have displayed excellent potential applications in the field of gas storage,^4,5^ catalysis,^6,7^ fluorescence,^8,9^ and magnetism.^10,11^ The general strategy to design CPs is to use organic ligands to connect metal ions or clusters into polynuclear clusters or polymeric networks. The choice of metal ions and organic linkers with specific coordination preferences is crucial to construct CPs through serendipitous synthesis or rational design. In this context, multi carboxylate ligands have been among the most extensively used ligands owing to their versatile and diverse in both coordination chemistry and magnetism.^12–15^ For instance, the carboxylate groups can bind two or more metal ions in various bridging modes, such as *syn*–*syn*, *syn*–*anti*, and *anti*–*anti*. In addition, they can form strong hydrogen bonds in favor of the formation of extended supramolecular structures and reinforcement the coordination networks. More importantly, they can efficiently transmit ferromagnetic (FM) or antiferromagnetic (AFM) coupling, depending on the bridging mode and the metal ion. Among them, the pyridyl-polycarboxylate ligands, such as pyridyl-di and tricarboxylates, have been largely explored, obtaining a variety of MOFs with fascinating structures and properties.^14,15*b*,*c*^ For instance, Zhao *et al.*^14*c*^ have synthesized a UiO type MOF based on 2,2′-bipyridine-5,5′-dicarboxylate (bpdc) ligand. This MOF exhibits high storage capacities for H_2_, CH_4_ and CO_2_. In the previous paper, we described the series of Zn(ii), Co(ii), Ni(ii), Mn(ii)^15*b*^ and Ln(iii)-CPs^15*c*^ based on 4-(3,5-dicarboxylphenyl)-2-methylpyridine (H_2_L) ligands. These CPs exhibit the various topologies with interesting magnetic properties and luminescence properties. Encouraged by above results, we decided to explore a new rigid pyridyl-tricarboxylates ligand, 4-(3,5-dicarboxylphenyl) picolinic acid (Scheme 1), which still has been unexplored. The π-conjugated rigid backbone and the tetratopic connectivity would lead to some new features in the coordination chemistry. Here we present the synthesis, structures and magnetic properties of four isomorphic CPs: [M_3_(L)_2_(H_2_O)_6_]·4H_2_O [M_3_ = Mn_3_ for 1; Co_3_ for 2; Ni_3_ for 3; Co_1.01_Ni_1.99_ for 4; H_3_L = 4-(3,5-dicarboxylphenyl) picolinic acid]. These CPs consist of 3D frameworks, in which 2D layers with single *syn*–*anti* carboxylate bridges are interlinked by the L spacers. CPs 1 and 2 display AFM couplings, while the FM interaction was found in CP 3 and the competition effect of FM and AFM interactions exists in CP 4.

## Experimental section

### Materials and physical measurements

All the starting chemicals including 4-(3,5-dicarboxylphenyl) picolinic acid (H_3_L) were used as received. The Fourier transform infrared spectra were recorded in the range 500–4000 cm^−1^ on a NEXUS 670 FT-IR spectrometer using the KBr pellets. Elemental analysis (EA) was determined on an Elementar Vario E1 III analyzer. Inductively coupled plasma (ICP) analysis was performed on a Optima 7300 DV. Powder X-ray diffraction (PXRD) was recorded on a Bruker D8-ADVANCE diffractometer equipped with Cu-target tube at a scan speed of 1 min^−1^. Magnetic measurements were carried out on a Quantum Design SQUID MPMS-5 magnetometer. Diamagnetic corrections were made with Pascal's constants. Thermogravimetric analyses were performed using a Mettler Toledo TGA/SDTA851 instrument at a heating rate of 5 °C min^−1^ under the N_2_ atmosphere.

#### Synthesis of [Mn_3_(L)_2_(H_2_O)_6_]·4H_2_O (1)

MnCl_2_·4H_2_O (0.036 g, 0.1 mmol) and H_3_L (0.029 g, 0.1 mmol) were mixed in water (4 mL) and stirred for 30 min. The solution was sealed in a Teflon-lined stainless steel vessel (25 mL), and heated at 100 °C for 3 days. Colorless microcrystals of 1 were obtained in a yield of 56% based on Mn. Our efforts to obtain single crystals suitable for X-ray crystallographic analysis is in vain. However, the PXRD measurement of 1 indicates that 1 is isomorphic with 2 (Fig. S1†). Anal. calc. for C_28_H_32_Mn_3_N_2_O_22_: C, 36.80; H, 3.53; N, 3.07. Found: C, 36.83; H, 3.54; N, 3.06%. IR (KBr, cm^−1^): 3371s, 1628s, 1576s, 1456m, 1364s, 1267m, 1066m, 1014m, 905m, 860m, 779m, 722m, 654m.

#### Synthesis of [Co_3_(L)_2_(H_2_O)_6_]·4H_2_O (2)

The preparation of CP 2 is similar to that of 1, except that MnCl_2_·4H_2_O was replaced by CoCl_2_·6H_2_O (0.10 mmol, 0.024 g). Red block crystals were isolated in a yield of 65% based on Co. Anal. calc. for C_28_H_32_Co_3_N_2_O_22_: C, 36.33; H, 3.49; N, 3.03. Found: C, 36.35; H, 3.51; N, 3.04%. IR (KBr, cm^−1^): 3279s, 1627s, 1588s, 1458m, 1372s, 1269m, 1066m, 1016m, 905m, 866m, 774m, 722m, 648m.

#### Synthesis of [Ni_3_(L)_2_(H_2_O)_6_]·4H_2_O (3)

The preparation of CP 3 is similar to that of 1, except that MnCl_2_·4H_2_O was replaced by NiCl_2_·6H_2_O (0.10 mmol, 0.024 g). Green block crystals were isolated in a yield of 65% based on Co. Anal. calc. for C_28_H_32_Ni_3_N_2_O_22_: C, 36.44; H, 3.50; N, 3.04. Found: C, 36.45; H, 3.52; N, 3.06%. IR (KBr, cm^−1^): 3388s, 1627s, 1588s, 1446m, 1376s, 1267m, 1072m, 1016m, 903m, 868m, 778m, 719m, 658m.

#### Synthesis of [Co_1.01_Ni_1.99_(L)_2_(H_2_O)_6_]·4H_2_O (4)

The preparation of CP 4 is similar to that of 1. The reaction of CoCl_2_·6H_2_O (0.05 mmol, 0.012 g), NiCl_2_·6H_2_O (0.05 mmol, 0.012 g), H_3_L (0.1 mmol, 0.029 g), H_2_O (4 mL) at 100 °C for 3 days. Grey green block crystals were isolated in a yield of 60% based on Co or Ni. Anal. calc. for C_28_H_32_Co_1.01_Ni_1.99_N_2_O_22_: C, 36.40; H, 3.49; N, 3.03. Found: C, 36.42; H, 3.51; N, 2.99%. IR (KBr, cm^−1^): 3386s, 1626s, 1590s, 1447m, 1379s, 1265m, 1070m, 1017m, 907m, 865m, 776m, 721m, 654m.

### Crystal structure analysis

Diffraction intensity data for 2, 3 and 4 was collected at 298 K on a Bruker Apex II CCD area detector equipped with graphite-monochromated Mo Kα radiation (*λ* = 0.71073 Å). Empirical absorption corrections were applied using the SADABS program.^16^ The structures were solved by the direct method and refined by the full-matrix least-squares method on *F*^2^ using SHELXTL-2014 program, with all non-hydrogen atoms refined with anisotropic thermal parameters.^17^ All of the hydrogen atoms attached to carbon atoms were placed in calculated positions and refined using the riding model, and the water hydrogen atoms were located from the difference maps. However, the hydrogen atoms of the guest water molecules (O10, O11) in CP 3 and 4 cannot be located because of the diffraction data of limited quality. These hydrogen atoms have been included in the formulas. All calculations were carried out with the SHELXTL crystallographic software. A summary of the crystallographic data, data collection, and refinement parameters for compound 2, 3 and 4 is provided in Table 1. CCDC reference numbers 1811142–1811144.

**Table 1:** Crystal data and structure refinements for CPs 2–4

CPs	2	3	4
Formula	C_28_H_32_Co_3_N_2_O_22_	C_28_H_32_Ni_3_N_2_O_22_	C_28_H_32_Co_1.01_Ni_1.99_N_2_O_22_
Mr	925.35	924.69	924.88
Crystal system	Monoclinic	Monoclinic	Monoclinic
Space group	*P*2_1_/*c*	*P*2_1_/*c*	*P*2_1_/*c*
*a* [Å]	17.0967(15)	16.6233(15)	16.699(4)
*b* [Å]	13.6362(13)	13.4654(13)	13.510(3)
*c* [Å]	7.1320(7)	7.1298(7)	7.1625(18)
*α* [°]	90	90	90
*β* [°]	97.993(4)	100.442(4)	100.626(9)
*γ* [°]	90	90	90
*V* [Å^3^]	1646.6(3)	1569.5(3)	1588.1(6)
*Z*	2	2	2
*ρ*_calcd_ [g cm^−3^]	1.866	1.957	1.934
*μ* [mm^−1^]	1.594	1.887	1.865
*F*(000)	942	948	948
Unique reflns	2916	2768	2805
GOF on *F*^2^	1.113	1.047	1.057
*R*_int_	0.0546	0.0701	0.0809
*R*_1_ (*I* > 2*σ*(*I*))	0.0370	0.0436	0.0444
w*R*_2_ (all data)	0.0978	0.1141	0.1185

## Results and discussion

### Synthesis and FT-IR spectra

CPs 1–4 were all synthesized by the hydrothermal reaction of MCl_2_·*n*H_2_O (*n* = 4 or 6) and H_3_L. CPs 2–4 were obtained in a Teflon-lined stainless steel vessel under 100 °C for 3 days. But the same procedure for Mn(ii) yielded microcrystalline products. We explore different ratios of Co/Ni source compared to the final ratios of Co/Ni in the framework. Only one pure ratios of Co/Ni, *ca.* 1.01 : 1.99, have been observed. These CPs exhibit similar characteristic asymmetric (*ν*_as_) and symmetric (*ν*_s_) absorptions of the carboxylate groups. The *ν*_as_(COO) and *ν*_s_(COO) vibrations appear as strong bands at about 1627 and 1450 cm^−1^, respectively.

### PXRD and TGA

The phase purity of the bulk materials of CPs 1–4 was confirmed by PXRD experiments (Fig. S1†). The PXRD patterns of samples in CPs 2–4 are in good agreement with those simulated from experimental data. And the PXRD patterns of CP 1 is also in good agreement with those of CPs 2–4, suggesting 1 is isomorphous with 2–4. This is also confirmed by FT-IR spectra and elemental analyses. The thermal stability of CPs 1–4 was measured under an air atmosphere (Fig. S2†). Four CPs showed similar weight loss curves. They show the weight loss of 19.30% (calcd. 19.70%) for CP 1, 19.25% (calcd. 19.45%) for CP 2, 19.10% (calcd. 19.46%) for CP 3, 19.19% (calcd. 19.46%) for CP 4 in the range of 23–320 °C, corresponding to the loss of all water molecules. The main frameworks of CPs 1–4 begin to collapse from 320 °C.

### Crystal structures

**Fig. 1:**
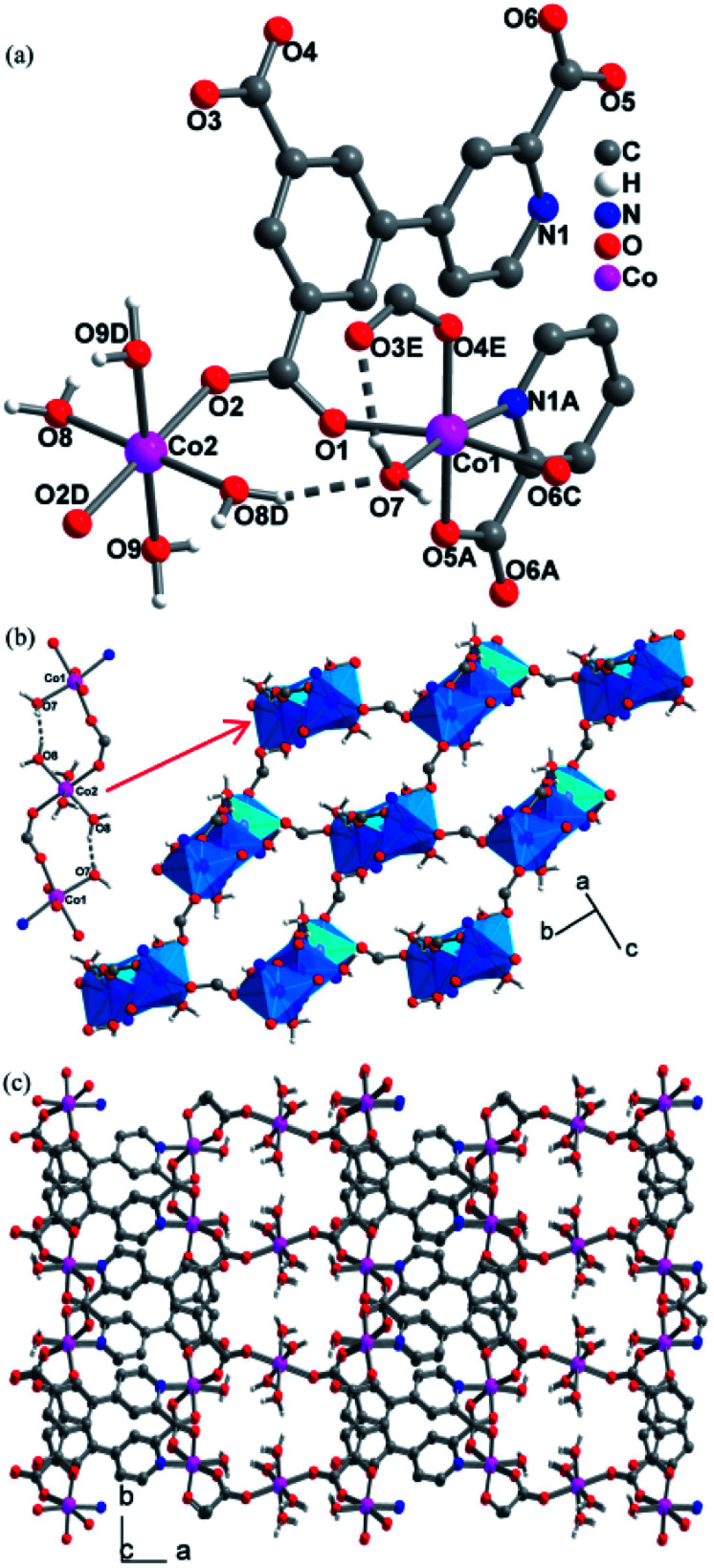
The structure of CP 2: (a) the coordination environment of Co(ii) ions; (b) the 2D (4,4) Co(ii)-carboxylate layer and the intralayer hydrogen bonds; (c) the 3D structure. (Symmetry codes: (A) *x*, −*y* + 1/2, *z* − 1/2; (B) −*x* + 3, −*y* + 1, −*z* + 1; (C) −x + 3, *y* + 1/2, −*z* + 1/2; (D) −*x* + 4, −*y* + 1, −*z* + 1; (E) *x*, −y + 1/2, *z* + 1/2; (F) *x*, −*y* + 1/2, *z* − 1/2).

Single crystal X-ray structure analyses revealed that CPs 2, 3 and 4 are isomorphous, crystallizing in the *P*2_1_/*c* space group and exhibiting 3D frameworks in which M(ii)-carboxylate layers are interlinked by the L ligands. The relevant parameters are summarized in Table S1.† The structure of 2 is described here in details. The asymmetric unit consists of one and a half of Co(ii) ion, one L ligand, three coordinated H_2_O molecules and two guest H_2_O molecules. As shown in Fig. 1a, there are two crystallographically independent Co(ii) ions in 2 (Co1 and Co2). Co1 assumes a distorted octahedral [NO_5_] geometry ligated by one pyridyl nitrogen atom (N1A) and four carboxylate oxygen atoms (O1, O4E, O5A, and O6C), and a H_2_O molecule (O7). Co2 resides at an inversion center and is coordinated in the *trans*-octahedral geometry by four H_2_O molecules (O8, O8D, O9 and O9D) at the equatorial plane and two equivalent carboxylate oxygen atoms (O2 and O2D) at axial positions. The Co–N/O bond distances for both Co1 and Co2 fall in the range of 2.158(3)–2.013(3) Å. The Co–O/N distances are comparable to those previous Co(ii) CPs based on the similar pyridine–tricarboxylate ligands.^15*d*,*e*^ Adjacent Co1 and Co2 atoms are bridged by single μ-*syn*, *anti*-carboxylate bridges. Through such bridges, each Co2 is linked to two equivalent Co1 atoms to form a centrosymmetric linear trinuclear motif (Fig. 1b). In the trinuclear motif, the Co1⋯Co2 distance separated by the single μ-*syn*, *anti*-carboxylate bridge are 5.372(9) Å. It is notable that the trinuclear motif is reinforced by the hydrogen bonds between the coordinated H_2_O molecules (O7 and O8) with O8–H8C⋯O7 = 168.5(1)°, H⋯O = 1.772(3) Å, O7⋯O6 = 2.678(4) Å. The hydrogen bonds set up triatomic O–H⋯O bridges between Co(ii) ions, and thus, it can be thinked that the adjacent Co(ii) ions are linked by the double [(μ-*syn*–*anti*-COO)(O–H⋯O)] bridges. Each trinuclear motif is connected to four neighboring ones through the other single μ-*syn*–*anti*-carboxylate bridges with the Co⋯Co separating of 5.651(1) Å, giving a 2D layer parallel to the *ab* plane (Fig. 1b). Topologically, each trinuclear motif serves as a 4-connecting node, which reduces to the (4,4) topology. The layers are pillared into a 3D framework by the L ligands (Fig. 1c).

The L ligand serves as a hexadentate bridge ligand and each ligand binds five Co(ii) ions through its three carboxylate groups and one pyridyl nitrogen atom in different modes: the carboxylate attached to the pyridyl ring and one pyridyl nitrogen atom (N1) connects with two Co(ii) ions, which induces a *syn*–*anti* coordination fashion for the carboxylate group, with the Co1F–O6–C14–O5 and Co1–O5–C14–O6 torsion angles being 53.7(5)° and 170.6 (3)°, respectively, and another carboxylate group also binds two Co(ii) ions in a *syn*–*anti* fashion, while third carboxylate group connects with one Co(ii) ion in monodentate fashion. The structures in 2 have two lattice water (O10, O11) per asymmetric unit that forms rich hydrogen bonds with carboxylate oxygen atoms and coordinated H_2_O molecules. The relevant hydrogen bonding parameters are given in Table S2.† Each lattice H_2_O molecule (O10, O11) donates its two hydrogen atoms to form two hydrogen bonds with carboxylate oxygen atoms (O1, O5, O3) and coordinated H_2_O molecule (O9), and each O10 and O11 atom also acts as a bifurcate hydrogen acceptor to interact with four different coordinated H_2_O molecules (O7, O8, O9, O9[−*x* + 1, *y* + 1/2, −*z* + 1/2]). Two tetrahedral geometries around O10 and O11 are formed by the four hydrogen bonds. Moreover, the coordinated H_2_O molecule (O8) uses its two hydrogen atoms to form one hydrogen bond with coordinated H_2_O molecule (O9). Five H_2_O molecules (O7, O8, O9, O10, O11) constitute a hydrogen-bonding ten-membered ring [graph set *R*_5_^5^(10)^18^ (Fig. S3†)].

### Magnetic properties

**Fig. 2:**
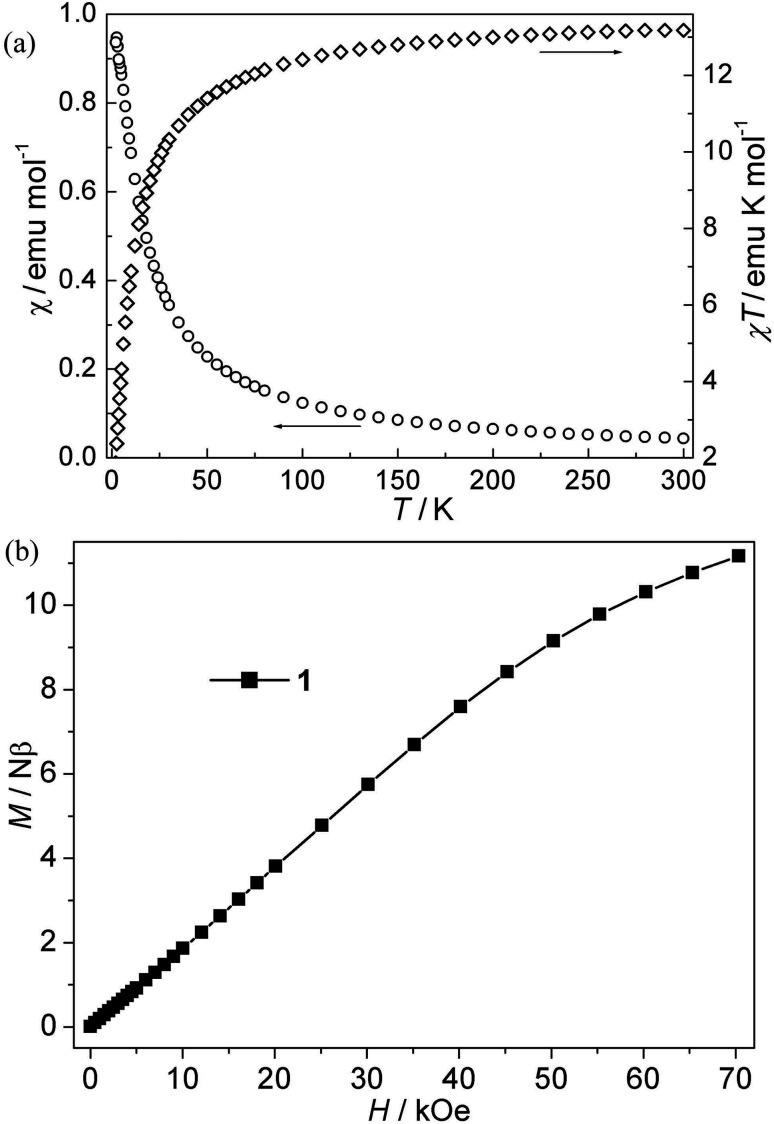
(a) Plots of *χ vs. T* and *χT vs. T* for 1; (b) isothermal magnetization for 1.

**Fig. 3:**
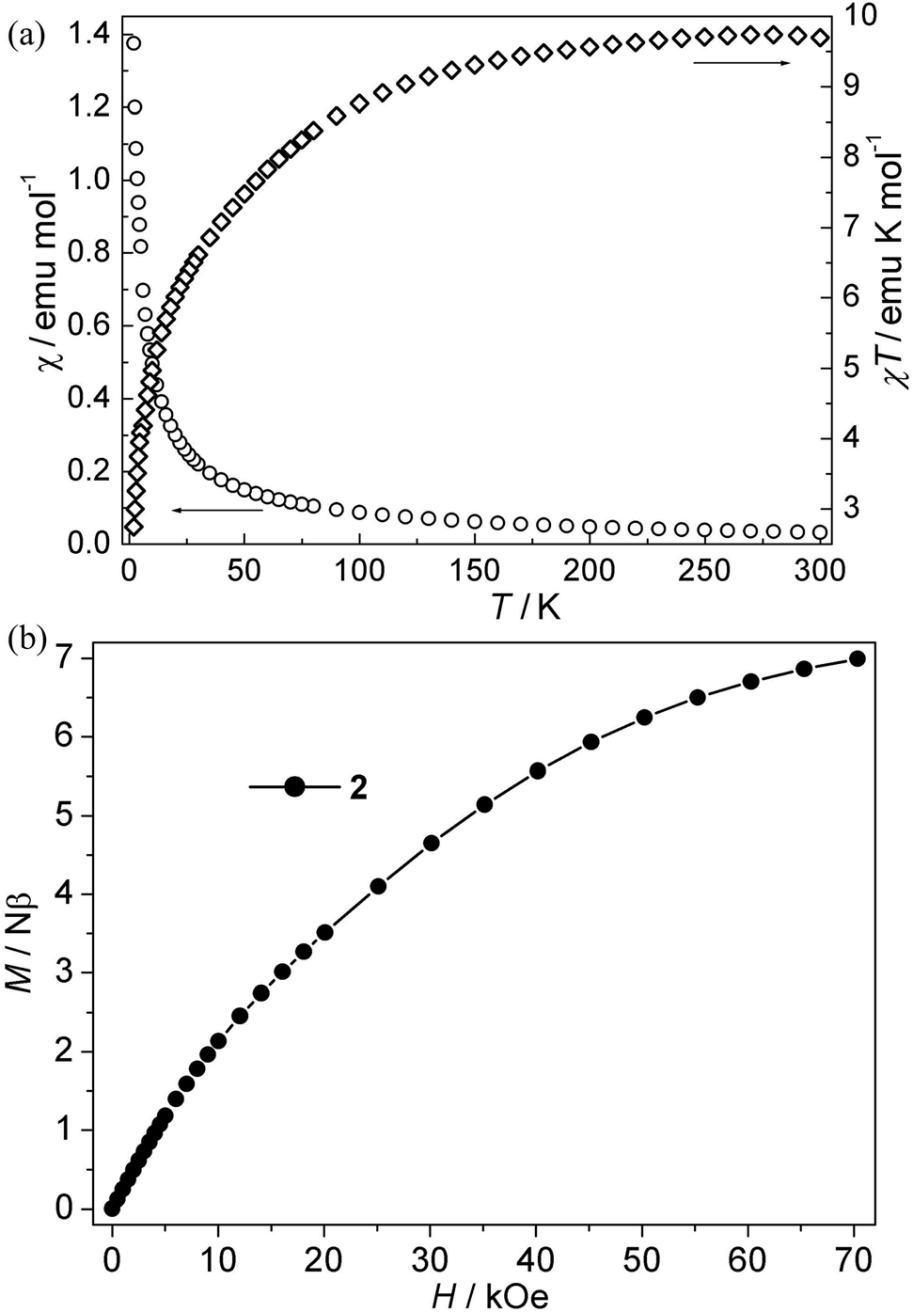
(a) Plots of *χ vs. T* and *χT vs. T* for 2; (b) isothermal magnetization for 2.

**Fig. 4:**
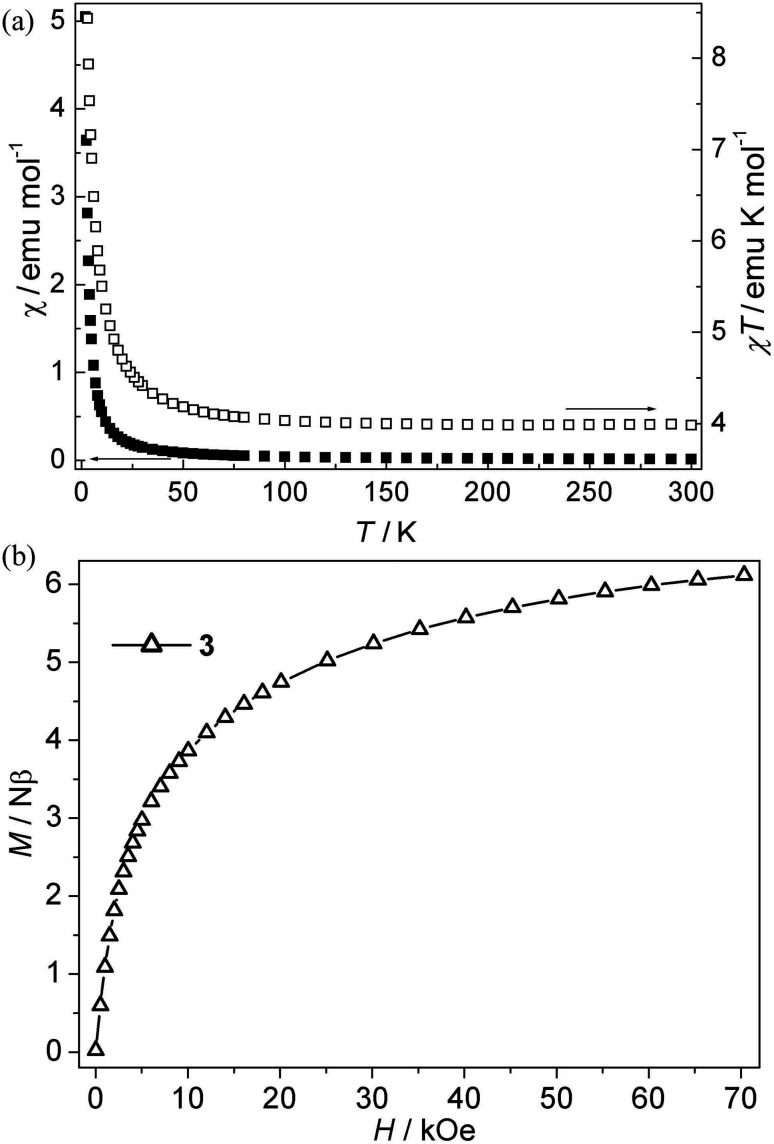
(a) Plots of *χ vs. T* and *χT vs. T* for 3; (b) Isothermal magnetization for 3.

**Fig. 5:**
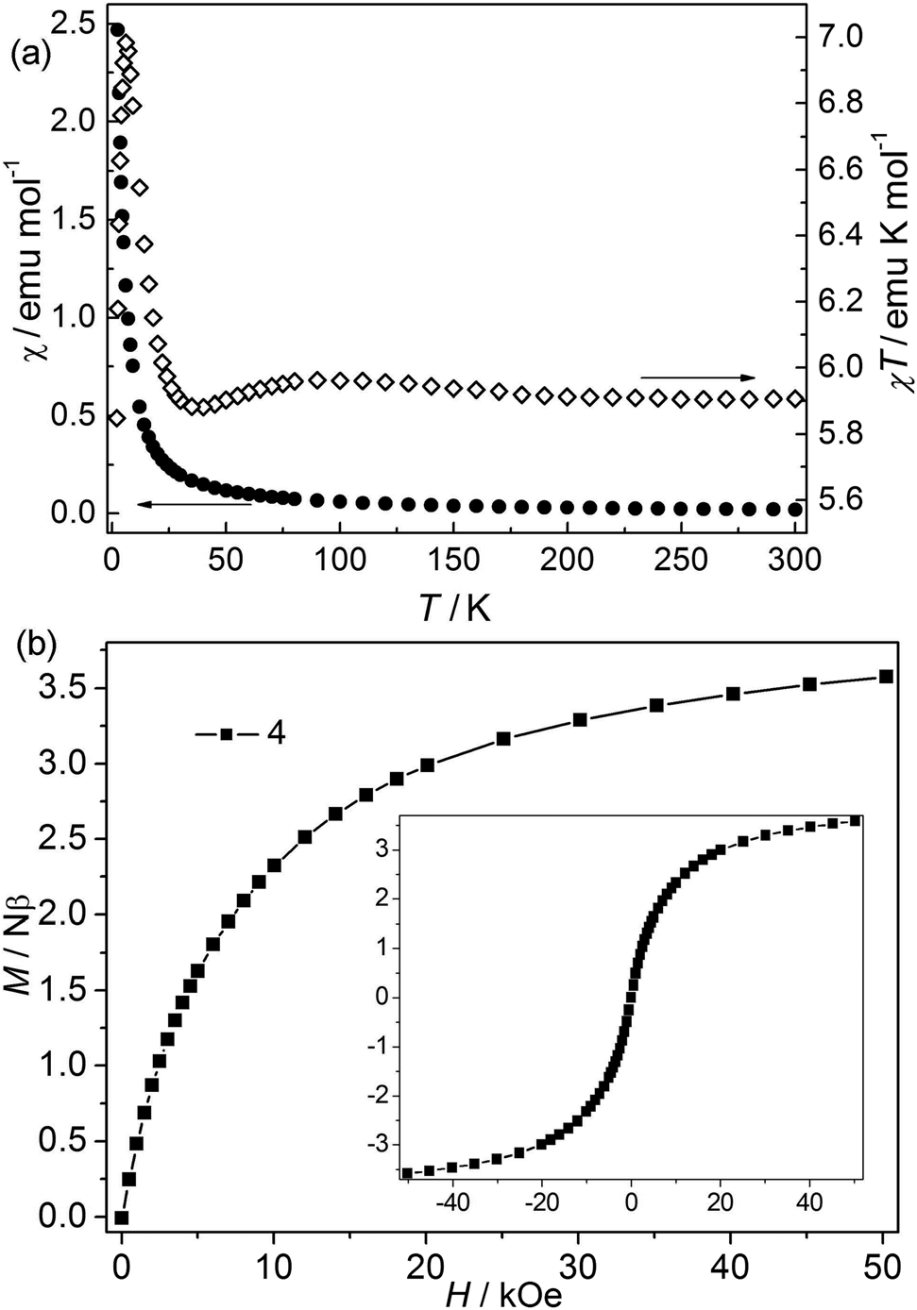
(a) *χ* and *χT vs. T* plots of 4 at 1 kOe; (b) isothermal magnetization for 4 at 2 K.

CPs 1–4. The magnetic susceptibility of 1–4 was performed on polycrystalline samples under 1 kOe in the range of 2–300 K (Fig. 2–5). For Mn(ii) CP 1, the *χT* values per Mn_3_ at 300 K is about 13.15 emu K mol^−1^, comparable with the value expected for three magnetically isolated Mn(ii) ions (13.13 emu K mol^−1^). Upon cooling, the *χT* value decreases continuously, which indicates the occurrence of antiferromagnetic interaction in this compound. However, the *χ* value increases monotonically (Fig. 2a). The data follow the Curie–Weiss behavior above 70 K with *C* = 13.93 emu K mol^−1^ and *θ* = −9.45 K. The negative *θ* value further confirms the overall antiferromagnetic behaviors between Mn(ii) ions. Due to the lack of an appropriate formula for such system, the *J* values (*J* is the interaction between Mn(ii) ions) can not be quantitatively estimated. However, magneto-structural comparisons with previous compounds indicate that the μ-*syn*, *anti*-carboxylate bridges induce antiferromagnetic interactions between Mn(ii) ions.^19,20^ In addition, the antiferromagnetic couplings is also confirmed by the isothermal magnetization measurement at 2 K (Fig. 2b). The magnetization curve is increases quasilinearly with the field lifted and the value (11.13 Nβ) at 70 kOe is much lower than the saturation value.

Co(ii) CP 2. As is shown in Fig. 3a, the *χT* values per Co_3_ at room temperature for this compound is about 9.72 emu K mol^−1^, which is much larger than the spin-only value for three isolated uncorrelated Co(ii) ions with *S* = 3/2. However, this is typical of pseudo-octahedral Co(ii) systems with a significant contribution from unquenched orbital momentum. Upon cooling, the *χT* value decreases continuously, while the *χ* value increases gradually, indicating the antiferromagnetic interaction between Co(ii) ions. The *χ*^−1^*vs. T* curve follow the Curie–Weiss law above 140 K with *C* = 10.20 emu K mol^−1^, *θ* = −13.60 K. It is noted that the negative *θ* value is not necessary for the antiferromagnetic interactions because of the effect of first-order spin-orbital coupling in Co(ii) system. In fact, the *χT* value of 2 is only 0.85 × 3 emu K mol^−1^ per Co(ii) at 2 K, which is very lower than that expected for the effective ground Kramer's doublet with a typical *g*_av_ value of 4.3, indicative of antiferromagnetic couplings.^21^ Moreover, isothermal magnetizations were also measured in the range of 0–70 kOe at 2 K. As is shown in Fig. 3b, the magnetization rises much more rapidly as the filed was lifted from zero and the magnetization value of 6.97 Nβ at 70 kOe is far from saturation, which supports the dominant antiferromagnetic coupling. In spite of no appropriate molde to quantitatively analyze the magnetic coupling in this system, However, magneto-structural comparisons with the related reports about Co(ii) compounds indicate that the μ-*syn*–*anti*-carboxylate bridges induce antiferromagnetic interactions between Co(ii) ions. This magnetic data results are in accordance with that in literature.^19*a*,22^

Ni(ii) CP 3. As is shown in Fig. 4a, the *χT* value per Ni_3_ at room temperature for this compound is about 4.00 emu K mol^−1^, which is much larger than the spin-only value (3.00 emu K mol^−1^) for three isolated Ni(ii) ions with *S* = 1 spins. Upon cooling, the *χT* value increases slowly up to 4.59 emu K mol^−1^ from 300 K to 20 K, and then the value rapidly increases to a maximum value of 10.06 emu K mol^−1^ at 2.0 K, which indicates that the ferromagnetic interaction is induced by single *syn*–*anti*-carboxylate bridges between Ni(ii) ions. The *χ*^−1^*vs. T* curve follow the Curie–Weiss law above 10 K with *C* = 3.94 emu K mol^−1^, *θ* = 2.85 K. The Curie constant (*C*) is in accordance with the theoretical value of an *S* = 1 free ion (*C* = 1 emu K mol^−1^ for three isolated Ni(ii)). The positive *θ* value suggests ferromagnetic interactions between Ni(ii) ions. The ferromagnetic coupling is also supported by the isothermal magnetization measurements at 2 K (Fig. 4b). The magnetization first sharply increases and then slowly increases to 6.10 Nβ at 70 kOe. The value (*J*) of 3 can not be given because of the lack of an appropriate model for such system. However, the magnetic couplings are consistent with the previous compounds with the *syn*–*anti* carboxylate bridge mediating ferromagnetic interactions.^22*f*,23^ Nevertheless, magneto-structural studies on Ni(ii) compounds with the *syn*–*anti* carboxylate bridges are scarce.

Bimetallic CP 4. The *χT* value at 300 K is about 5.9 emu K mol^−1^, falling within the values of 2 (9.72 emu K mol^−1^) to 3 (4.00 emu K mol^−1^) (Fig. 5).^24^ The value is higher than the spin-only values for three isolated high-spin Co(ii) (5.64 emu mol^−1^ K) and Ni(ii) (3.00 emu mol^−1^ K). However, it is consistent with the first-order orbital moment contribution of Co(ii) ions in the octahedral field. Upon cooling, the *χ* value increases continuously, while the *χT* value first increases slightly to a round maximum at about 80 K and then slowly decreases to a minimum at 5.87 K, after that it increases sharply to a maximum of 6.99 emu K mol^−1^ at 4.27 K, and finally drops to 5.85 emu K mol^−1^ at 2.0 K. The data follow the Curie–Weiss law above 10 K with *C* = 5.90 emu K mol^−1^ and *θ* = 0.59 K. The increase of *χT* value in the range of 80–300 K suggests a ferromagnetic interaction in this structure. The low-temperature behavior of *χT vs. T* may be attributed to the cooperation or competition of several effects such as spin-orbital coupling and magnetic interactions between the different metal(II) ions. Isothermal measurements (Fig. 5) revealed that the molar magnetization rises continuously to 3.58 Nβ at 50 kOe with increased field, which is far from saturation, supporting the competitive effects of the magnetic coupling and the anisotropy. No hysteresis loops were observed at 2.0 K in CP 4 (Fig. 5 inset). The field-cooled (FC) and zero-field-cooled (ZFC) curves are measured at 20 Oe from 2 to 20 K (Fig. S4†). The two curves are identical, indicating no occurrence of the phase transition or ordering. This is also confirmed by the thermal ac susceptibility curves (Fig. S5†). The ac curves show the frequency independent behaviors. The real component (*χ*′) shows no maximum, meanwhile, no imaginary signal (*χ*′′) is observed.

## Discussion

Generally speaking, the *anti*–*anti* carboxylate bridges induces antiferromagnetic interactions between paramagnetic metal ions owing to the geometry of the magnetic orbital. Moreover, the double *syn*–*syn* carboxylate bridges also usually induce antiferromagnetic couplings, while the *syn*–*anti* carboxylate bridges may transmit weak antiferro- or ferromagnetic interactions.^25^ After the literature research, we found that the single carboxylate bridges in the Mn(ii) compounds always induce antiferromagnetic interactions. The magnetic coupling of CP 1 is antiferromagnetic, which is in accord with the previous compounds in literature.^19,20^ However, only a few Mn(ii) compounds shows the spin-canted ferromagnetic ordering above 2 K. In the context, many Co(ii)-carboxylate compounds have been reported. However, the most of them are double carboxylate bridged. Thus, the Co(ii) species with single *syn*–*anti* carboxylate bridges are still lacking. Moreover, the magnetic analysis for octahedral Co(ii) is complicated due to its single-ion effects. Therefore, it is difficult to extract magnetostructural correlations. Some literatures survey suggest that single *syn*–*anti* carboxylate bridges between Co(ii) indued antiferromagnetic couplings. In 2, the adjacent Co(ii) ions bridged through the single *syn*–*anti* carboxylate bridges is a new examples in this series. Ni(ii) CP 3 with the single *syn*–*anti* carboxylate bridges are much less than Co(ii) and Mn(ii) species with the similar bridges in the literature. Only few exceptions of this series are known in which single *syn*–*anti* carboxylate bridges all transmit ferromagnetic interactions between Ni(ii) ions. The magnetic behaviors in bimetallic CP 4 depend mainly upon the random spin distribution from their parent CPs 2 and 3. Co(ii) CP 2 show a typical antiferromagnetic coupling with antiparallel arrangement of spins, while Ni(ii) CP 3 with parallel arrangement of spins show a typical ferromagnetic coupling. Thus, when the second spins, Ni(ii) ions are incorporated into Co(ii) system, the Co(ii) ions would be substituted by Ni(ii) ions. Therefore, in CP 4, there are two competing effects, including the decreasing anisotropy. Such effect is similar to that in previous bimetallic Co(ii)–Ni(ii) system.^24^ Although CPs 1–4 are isostructural, magnetically, they exhibit the interesting different magnetic behaviors through the (*syn*–*anti*-COO) single bridge: 1 and 2 are antiferromagnetic, 3 is ferromagnetic and 4 is complicated magnetic behaviors. It is noted that so far, no CPs based on such M(ii)-carboxylate layers have been reported. However, our results can be supported by previously reported M(ii)-carboxylate compounds (M = Co(ii), Mn(ii), Ni(ii)) with square-grid layers.

## Conclusions

In summary, we present four isomorphous CPs derived from a new pyridine-tricarboxylate ligands, in which adjacent M(II) ions are connected through single *syn*–*anti* carboxylate bridges into M(II)-carboxylate layers and then the layers are interlinked into 3D frameworks by the L spacers. The magnetic studies demonstrated that the single *syn*–*anti* carboxylate bridges transmit antiferromagnetic (AFM) interactions in the Mn(ii) and Co(ii) CPs while ferromagnetic (FM) in the Ni(ii) species. The bimetallic Co(ii)–Ni(ii) exhibit the competition effect of FM and AFM interactions. Although the theory of composition-effects is unclear, the experimental findings provide a good approach to improve magnetic behaviors by mixing metals. Thus, this work not only represents new structures based on M(ii)-carboxylate layers, but also represents the interesting magnetic behaviors are related to the nature of the metal(ii) ions.

## Conflicts of interest

There are no conflicts to declare.

## Supplementary Material

RA-008-C8RA01900B-s001

RA-008-C8RA01900B-s002
