# Supplementary material for: Four 3D coordination polymers based on layers with single syn–anti carboxylate bridges: synthesis, structures, and magnetic properties
Source: RSC Adv. 2018 Apr 16;8(25):14101–8. doi: 10.1039/c8ra01900b (PMC9079894; doi:10.1039/c8ra01900b)
Supplement: RA-008-C8RA01900B-s001 [file RA-008-C8RA01900B-s001.pdf]

Electronic Supplementary Material (ESI) for RSC Advances.
This journal is © The Royal Society of Chemistry 2018

Four 3D coordination polymers based on layers with single *syn-anti* carboxylate bridges: synthesis, structures, and magnetic properties

*Wei Gao[ab], Feng Liu[b], Xiu-Mei Zhang[*b], Jie-Ping Liu[b] and Qing-Yu Gao[*a]*

Table S1 Selected bond lengths (Å) and angles (°) for CPs **2-4**

	**2** (M = Co)	**3** (M = Ni)	**4** (M = CoNi)
M1-O1	2.149(2)	2.111(3)	2.119(3)
M1-O4A	2.023(2)	2.004(3)	2.010(3)
M1-O5B	2.087(2)	2.061(3)	2.070(3)
M1-O7	2.117(3)	2.071(3)	2.096(4)
M1-O6C	2.174(2)	2.108(3)	2.130(3)
M1-N1B	2.117(3)	2.056(3)	2.077(3)
M2-O2	2.107(2)	2.010(3)	2.021(3)
M2-O8	2.013(3)	2.072(6)	2.083(6)
M2-O9	2.158(3)	2.055(4)	2.074(5)
O4A-M1-O5B	168.70(10)	172.19(12)	171.89(12)
O4A-M1-O7	91.62(10)	94.02(13)	93.91(13)
O5B-M1-O7	98.37(10)	93.58(13)	94.00(13)
O4A-M1-N1B	92.65(10)	92.31(13)	92.67(12)
O5B-M1-N1B	78.16(10)	80.48(12)	79.86(12)
O7-M1-N1B	171.32(11)	168.86(14)	167.86(14)
O4A-M1-O1	92.49(10)	92.21(12)	92.20(12)
O5B-M1-O1	83.44(10)	86.25(13)	86.21(13)
O7-M1-O1	83.67(11)	88.22(14)	88.74(14)
N1B-M1-O1	103.67(10)	100.69(13)	101.21(13)
O4A-M1-O6C	98.39(10)	92.48(12)	92.87(12)
O5B-M1-O6C	86.87(10)	89.63(13)	89.32(13)
O7-M1-O6C	90.28(11)	87.50(14)	86.86(14)

N1B-M1-O6C	81.63(10)	83.08(13)	82.62(13)
O1-M1-O6C	167.70(9)	173.86(12)	173.50(12)
O8D-M2-O8	180.0	180.000(2)	180.000(2)
O8D-M2-O2	93.02(12)	89.0(2)	89.14(19)
O8-M2-O2	86.98(12)	91.0(2)	90.86(19)
O2-M2-O2D	180.00(13)	180.0(2)	180.00(19)
O8-M2-O9	91.10(18)	89.3(3)	89.7(2)
O8-M2-O9D	88.90(18)	90.7(3)	90.3(2)
O2-M2-O9D	83.63(11)	93.28(16)	93.49(17)
O2-M2-O9	96.37(11)	86.72(16)	86.51(17)
O9-M2-O9D	180.0	180.000(1)	180.000(2)

Symmetry codes: A x, -y+1/2, z-1/2; B -x+3, -y+1, -z+1; C -x+3, y+1/2, -z+1/2; D -x+4, -y+1, -z+1.

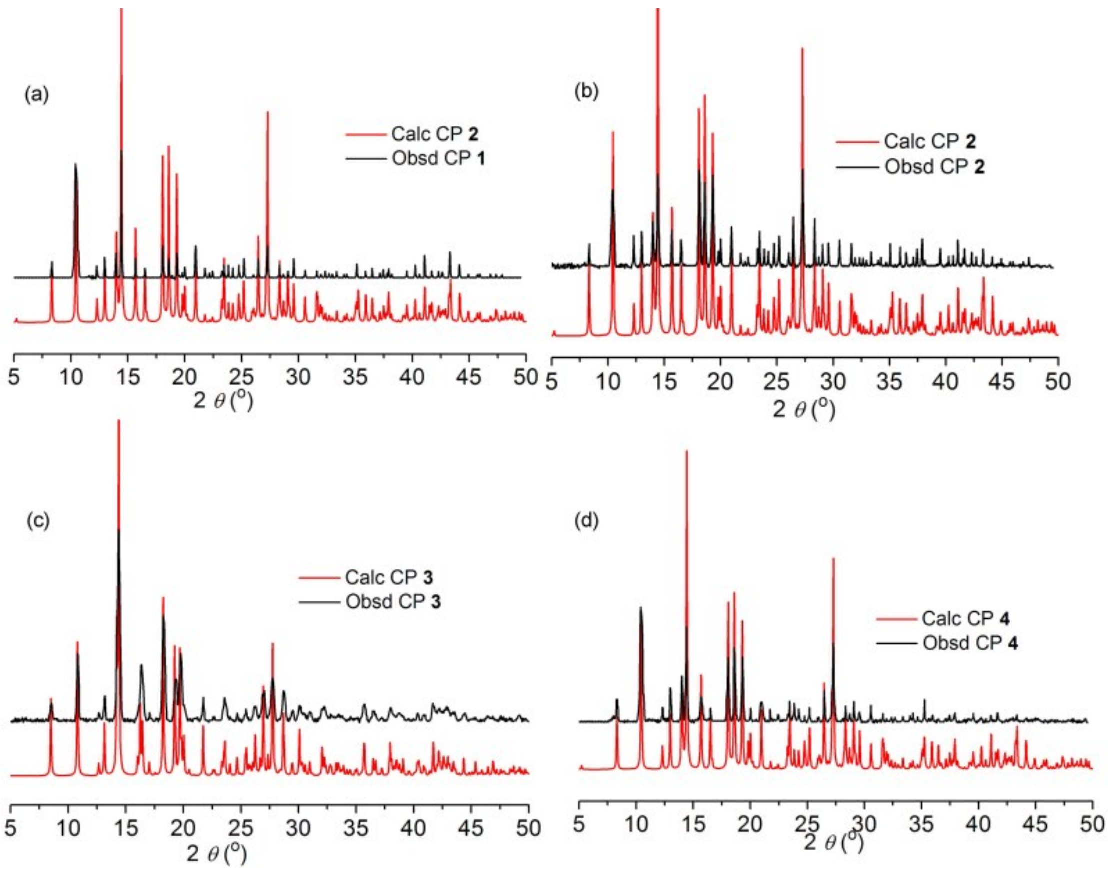

Fig. S1 Powder X-ray diffraction profiles (black) of CPs **1-4** together with a simulation from the single crystal data (red).

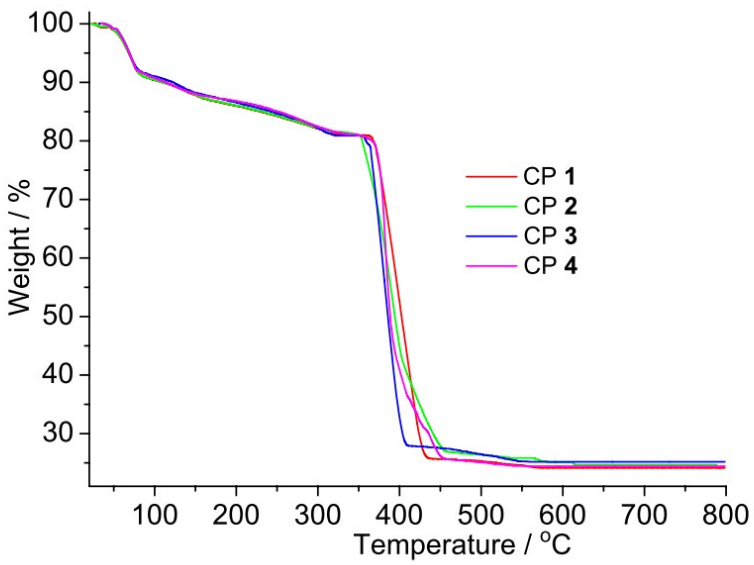

Fig. S2 TGA curves of CPs **1-4**.

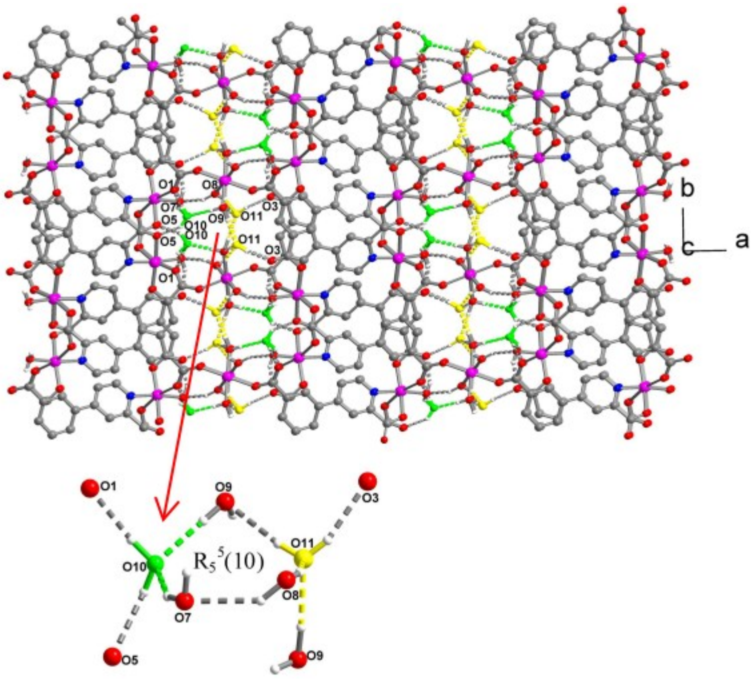

Fig. S3 The 3D structures with hydrogen bonding interactions i CP **2**.

Table S2 Hydrogen bond lengths (Å) and angles (°) for CP **2**[a].

D	A[Transformation]	d(D-H)	d(H···A)	d(D···A)	<(DHA)
O7	O10[x-1, y, z+1]	0.817	2.072	2.862	162.63
O8	O7 [-x, -y+1, -z+1]	0.847	2.035	2.852	162.01
O8	O11 [x-1, -y+3/2, z-1/2]	0.849	1.849	2.683	167.18
O9	O10[x-1, y, z]	0.880	1.760	2.635	172.33
O9	O11[-x+1, -y+1, -z+1]	0.869	2.027	2.894	174.84
O10	O1[x+1, y, z]	0.924	1.819	2.734	170.03
O10	O5[-x+2, y-1/2, -z+1/2]	0.849	2.145	2.824	136.71
O11	O3[x+1, y, z]	0.833	1.982	2.739	150.55
O11	O9[-x+1, y+1/2, -z+1/2]	0.845	2.013	2.846	168.64

[a] D, donor; A, acceptor.

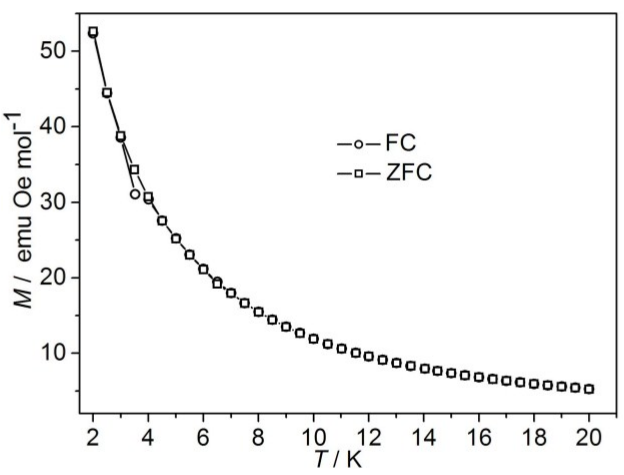

Fig. S4 The ZFC and FC curves of CP **4**.

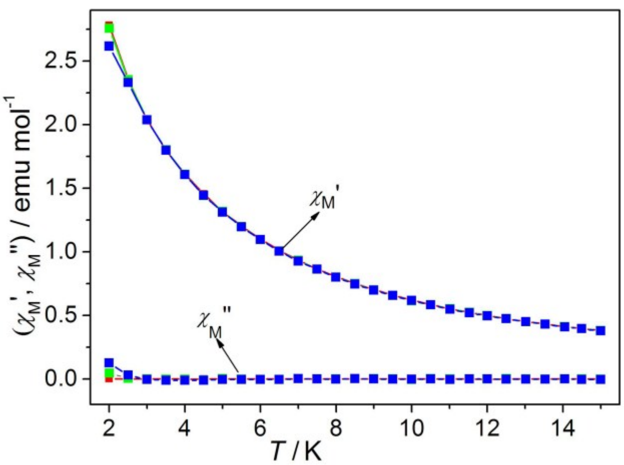

Fig. S5 $\chi_M{}'$ and $\chi_M{}''$ plots for CP **4** at frequencies of 10, 100, and 100 Hz with $H_{dc} = 0$ and $H_{ac} = 3.0$ Oe.
